# Supplementary material for: Chemical Composition and Biological Activities of Prangos ferulacea Essential Oils
Source: Molecules. 2022 Nov 1;27(21):7430. doi: 10.3390/molecules27217430 (PMC9657548; doi:10.3390/molecules27217430)
Supplement: Supplementary file 1 [file molecules-27-07430-s001.zip › molecules-1998056-supplementary.pdf]

Supplementary Data

Results

In order to determine or exclude a possible target towards which the essential oils of *P. ferulacea* flowers and leaves are directed, we performed fluorescence microscopy experiments against Gram-negative bacterium *E. coli,* as shown in **Figure S1.**

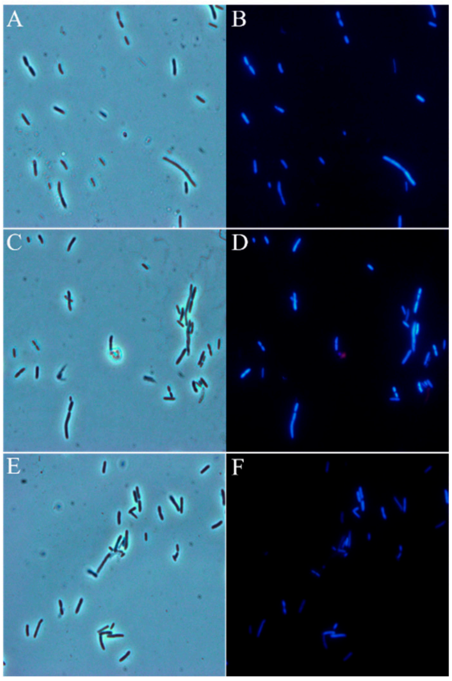

Figure S1: *E. coli* bacterial cells observed via optical microscopy and fluorescence microscopy. Untreated bacterial cells (A, B); cells treated with *P. ferulacea* flower oil at a concentration of 200 µg/mL (C, D); cells treated with *P. ferulacea* leaf oil at a concentration of 200 µg/mL (E, F).

The bacterial cells used as a control appeared intact and dark gray via optical, phase contrast microscopy (panels A), developing blue fluorescence in panel B. Bacterial cells treated with *P. trifida* essential oil from its flowers (panel C) and leaves (panel E) appeared not to be altered in shape or color, as shown in optical, phase contrast microscopy. The same samples developed a blue fluorescence signal due to DAPI staining, in comparison to the control, as shown in panels D and F. These bacterial cells do not show any membrane damage and did not develop any red fluorescence due to IP dye entry. Many essential oils, as the main mechanism of action, can alter bacterial membrane permeability, but further mechanisms underlying the antimicrobial activity are also possible. Indeed, Wang et al. demonstrate in their study that *Ginger* essential oil is able to inhibit the energetic expression of some genes related to bacterial metabolism, the cycle of tricarboxylic acid, proteins bound to the cell membrane and DNA metabolism [56]. Therefore, essential oils rich in terpenes have been shown to possess good antibacterial activity. However, the weak activity against Gram-negative microbes could be due to the presence of their double membrane that protects the Gram-negative bacteria from the effect of the oil's components [36].

Materials and Methods

Fluorescence Microscopy Image Acquisition

For dual staining, 100 μL of the bacterial culture of *Escherichia coli* (bacteria were grown to the mid-logarithmic phase) was incubated in the dark for 2 h at 37 *C with or without 200 μg/mL of *P. ferulacea* essential oils. After incubation, 10 μL of bacterial culture was mixed with DAPI solution (40 ,6-diamidino-2-phenylindole dihydrochloride; Sigma Aldrich, Milan, Italy) (1 μg/mL DAPI final concentration) and 20 μg/mL of PI (propidium iodide; Sigma Aldrich, Milan, Italy). The samples were analyzed using an Olympus BX51 fluorescence microscope (Olympus, Tokyo, Japan) with a DAPI filter (excitation/emission: 358/461 nm). Standard acquisition times were 1000 ms for DAPI/PI dual staining. Images were captured using an Olympus DP70 digital camera [57].
